# The cross-sectional correlation between the oxidative balance score and cardiometabolic risk factors and its potential correlation with longitudinal mortality in patients with cardiometabolic risk factors

**DOI:** 10.1186/s12889-024-18967-z

**Published:** 2024-05-30

**Authors:** Qiancheng Lai, Liu Ye, Jun Luo, Cheng Zhang, Qingchen Wu, Yue Shao

**Affiliations:** 1 Department of Cardiac Surgery, Sichuan Provincial People’s Hospital, University of Electronic Science and Technology of China, Chengdu, China; 2 Health Management Center, The First Branch, The First Affiliated Hospital of Chongqing Medical University, Chongqing, China; 3 Department of Cardiothoracic Surgery, The First Affiliated Hospital of Chongqing Medical University, NO. 1 Youyi Road, Yuzhong District, Chongqing, China

**Keywords:** Cardiometabolic risk factors, Mortality, NHANES, Oxidative balance score

## Abstract

**Background:**

This study analyzes the correlation between oxidative balance score (OBS), cardiometabolic risk factors (CMRFs), and mortality in individuals with CMRFs.

**Methods:**

Data were chosen from the National Health and Nutrition Examination Survey. The survey-weighted multivariable logistic regression models were implemented to explore the relationship between OBS and the risk of CMRFs. Then, Cox proportional hazard models were employed to estimate the impact of OBS on mortality in individuals with CMRFs.

**Results:**

Following multivariate adjustment, the subjects in the highest quartile exhibited a 46% reduction in the risk of CMRFs, a 33% reduction in the risk of diabetes, a 31% reduction in the risk of hypertension, and a 36% reduction in the risk of hyperlipidemia, compared with those in the lowest quartile. Furthermore, each 1-unit increase in OBS was remarkably negatively correlated with the prevalence of CMRFs, diabetes, hypertension, and hyperlipidemia. The correlation between OBS and CMFRs was found to be mediated by serum γ-glutamyltransferase (GGT) and white blood cells (WBC), and the mediation effect of GGT levels and WBC, accounting for 6.90% and 11.51%, respectively. Lastly, the multivariate Cox regression model revealed that elevated OBS, irrespective of whether it was treated as a categorical or continuous variable, exhibited a significant association with decreased mortality from all causes, cardiovascular disease, and cancer.

**Conclusions:**

An increased OBS might reflect a lower risk of CMRFs and a favorable prognosis for individuals with CMRFs. Moreover, WBC and GGT may play a potential mediating role between OBS and CMRFs.

**Supplementary Information:**

The online version contains supplementary material available at 10.1186/s12889-024-18967-z.

## Introduction

Cardiac metabolic risk factors (CMRFs) are considered risk factors for cardiovascular disease (CVD) development [1]. Hypertension, diabetes, and hyperlipidemia are recognized as the main metabolic risk factors, with prevalence rates varying between 10.5% and 31.1% [2–4]. These three factors collectively accounted for over 60% of global CVD-related mortality in 2010 [5]. Hence, the implementation of early preventive strategies targeting CMRFs can effectively result in a reduction in mortality rates.

Oxidative stress is one of the most fundamental mechanisms leading to the occurrence and development of CMRFs [6, 7]. The oxidative Balance Scale (OBS) is a comprehensive indicator representing the overall oxidative effects of prooxidants and antioxidants regarding dietary and lifestyle levels and associated with oxidative stress and inflammation [8, 9]. Smoking and alcohol consumption have been identified as potential oxidants, whereas exercise and a healthy dietary intake (dietary fiber, carotenoids, calcium, etc.) are deemed as potential antioxidants [10]. A previous small sample study based on the elderly population in China has established a significant association between lifestyle OBS and CMRFs [11]. Higher OBS has been demonstrated to be associated with a reduced risk of developing diabetes and hypertension [12–15]. Nevertheless, the existing data are subject to various limitations. For instance, the study performed by Arnor et al. [12] had a limited sample size of 317 participants, indicating a lack of generalizability. Kwon et al. [13] and Lee et al. [14] focused on middle-aged and elderly individuals in South Korea, as the dietary habits of the Western and Eastern populations are different. Therefore, further investigation is needed into the relationship between OBS and CMRFs in Western adults. Previous two studies have found that individuals with high OBS have a lower risk of all-cause mortality, including in cohorts of Spanish graduates and adults aged 45 and above in the “stroke belt” of the US [16, 17]. However, these studies did not explore the potential impact of OBS on mortality risk in individuals with CMRFs. Considering that CMFRs account for the majority of CVD-related deaths, exploring the correlation between OBS and mortality among subjects with CMRFs holds significant implications for tertiary preventive strategies.

To address this question, we utilized data derived from the National Health and Nutrition Examination Survey (NHANES). First, we conducted a cross-sectional study to explore the relationship between OBS and CMRFs and further evaluate the possible mediating roles of inflammation and oxidative stress indicators in this relationship. Next, we performed a longitudinal study to examine the correlation between OBS and mortality among subjects with CMRFs, including overall and disease-specific outcomes.

## Materials and methods

### Study population

The data used in this analysis were acquired from NHANES, which employed complex, stratified, and multistage probability sampling techniques to represent the health condition of the general population in the United States.

Part I: The present study included 101,316 participants spanning ten consecutive NHANES cycles (1999–2018). After excluding participants under 20 years old, pregnant women, and those without eligible OBS and CMRF data, a final sample size of 29,289 subjects was ultimately selected (Supplementary Fig. 1). Among them, 23,190 individuals were diagnosed with CMRFs. An analysis was implemented to evaluate the cross-sectional correlation between OBS and CMRFs.

Part II: We additionally included participants with a confirmed diagnosis of CMRFs, subsequently excluding 28 subjects due to loss of follow-up. As of December 31, 2019, out of 23,162 individuals with CMRFs, 3292 experienced all-cause mortality (Supplementary Fig. 1). Afterward, a longitudinal analysis was implemented to investigate the relationship between OBS and mortality.

### Definition of CMRFs and mortality

Part I: CMRFs were defined as hypertension, diabetes, hyperlipidemia, or any combination of the three conditions. The detailed definitions of hypertension, diabetes, and hyperlipidemia are provided in the Supplementary Methods.

Part II: Survival status and causes of death were ascertained by accessing the National Death Index file. ICD-10 was employed to determine the underlying causes of death among the participants, including mortality due to CVD (heart disease: I00–I09, I11, I13, I20–I51) and malignant neoplasms (C00–C97). The duration of follow-up was calculated by estimating person-years from the time of the interview until either the occurrence of death or December 31, 2019, whichever transpired first.

### OBS assessment

The calculation of OBS for each participant was derived from prior reports [18, 19]. Following this methodology, a comprehensive selection of 16 dietary and 4 lifestyle components were identified, all of which exhibit an association with oxidative stress. The specific evaluation methods for OBS are detailed in Supplementary Table 1. A greater OBS suggests that the antioxidative components exhibit a greater advantage compared to prooxidant components [20]. For detailed information on OBS evaluation, please refer to the supplementary methods.

### Measurement of inflammation and oxidative stress biomarkers

White blood cell (WBC) counts were measured using Beckman Coulter MAXM instruments in MECs with the Beckman Coulter method of counting and sizing, which were subsequently reported as ×1000 cells/µL [21, 22]. Gamma-glutamyl Transaminase (γ-GGT) was analyzed using a Hitachi Model 704 multichannel analyzer in NHANES 1999–2002, a Beckman Synchron LX20 in NHANES 2003–2008, a Beckman UniCel DxC800 Synchron analyzer in NHANES 2009–2016, and a Roche Cobas 6000 analyzer in NHANES 2017–2018.

### Covariates

The collection of baseline data was accomplished through conducting interviews and laboratory tests, including general information, dietary factors, and laboratory data [uric acid, estimated glomerular filtration rate (eGFR) and alanine aminotransferase (ALT) level]. General information included age, sex, ethnicity (including Black, White, Mexican, and others), educational attainment (ranging from below high school to above high school), marital status (separated and married), and poverty income ratio (1.3, 1.3–3.5, and ≥ 3.5). Dietary data included total energy, caffeine, and sodium intake, obtained through 24-hour dietary recall surveys. The measurement method for serum ALT, serum creatinine (SCr), and uric acid was the same as y-GGT. The eGFR was determined using the Chronic Kidney Disease Epidemiology equation [23], which included serum creatinine, age, sex, and ethnicity. For a detailed calculation formula for eGFR, please refer to the Supplementary Methods.

### Statistical analysis

All statistical analyses were conducted in compliance with NHANES analytical guidelines. No variable exhibited a missing data rate exceeding 10%, and multiple imputations were conducted to address missing values [24]. Continuous variables were expressed as the weighted mean ± standard error and analyzed using a one-way ANOVA. Categorical data were presented as weighted percentages, and group comparisons were assessed using the Chi-square test.

We implemented multivariable logistic regression analysis to analyze the relationship between OBS and CMRFs, and multiple Cox proportional hazards regression analysis to investigate the correlation between OBS and mortality due to all causes, CVD, and cancer. Three distinct models were constructed, with age, sex, and race/ethnicity adjusted in Model I; the remaining demographic information was incorporated in Model II; intakes of total energy, caffeine, sodium, uric acid, eGFR, and ALT were introduced in Model III. The OBS variable was constructed as a continuous variable and quartiles (Q1-Q4), with the lowest quartile serving as the reference category. In addition, potential correlations between dietary/lifestyle OBS, CMRFs, and mortality were analyzed. Restricted cubic spline (RCS) with 4 knots was utilized to visualize the dose-response relationship between OBS and the risk of CMRFs and mortality.

We employed mediation analysis to assess the potential mediating influence of GGT levels and WBC counts on the relationship between OBS and CMRFs. The mediation analysis was conducted using R package mediation and adjusting for confounding variables including general information, dietary factors, and laboratory data.

A series of sensitivity analyses were conducted to evaluate the robustness of the results. Firstly, given the relationship between different dietary patterns and CMRFs, the Dietary Approaches to Stop Hypertension (DASH) and Healthy Eating Index-2015 (HEI-2015) scores were introduced into the fully adjusted model. Secondly, subjects who experienced mortality within a two-year frame were excluded to mitigate the potential impact of reverse causality. Ultimately, we censored the follow-up at 10 years. We implemented all statistical analyses using R software (version 4.2.2). *P* values < 0.05 were considered statistically significant.

## Results

### Correlations between OBS and CMRFs

A total of 29,289 eligible participants were chosen for this analysis, aiming to examine the association between OBS and CMRFs. Among these participants, 4,233 individuals were diagnosed with diabetes, 11,736 individuals were diagnosed with hypertension, and 20,823 subjects were diagnosed with hyperlipidemia. Table 1 summarizes the main characteristics of participants based on OBS quartiles. The results indicated a higher level of OBS and a trend for a lower prevalence of CMRFs, diabetes, hypertension, and hyperlipidemia (all *P* < 0.001). As OBS increased, subjects were more likely to be male, non-Hispanic white, married, possess higher education levels, have a higher PIR, elevated levels of uric acid, and a higher intake of sodium and total energy. Notably, individuals with elevated levels of OBS exhibited increased ALT levels and caffeine consumption. Besides, differences were noted in age and eGFR levels among the groups. Supplementary Table 2 presents the individual components of OBS according to the OBS quartile. As OBS increased, there was a corresponding progressive increase in the consumption of dietary fiber, carotene, vitamin B12, vitamin C, vitamin E, vitamin B6, calcium, magnesium, zinc, copper, selenium, riboflavin, niacin, total folic acid, total fat, and iron. Likewise, there was a gradual increase in the duration of physical activity, whereas BMI and cotinine levels exhibited a gradual decrease (all *P*<0.001).

**Table 1:** Participant characteristics in NHANES 1999–2018 by quartiles of the OBS, weighted (Participants = 29,289/ CMRFs cases = 23,190)

Characteristics	Total	Q1	Q2	Q3	Q4	*P*
	*N* = 29,289	*N* = 8298	*N* = 7350	*N* = 6574	*N* = 7067	
Age (years)	46.29 (0.21)	45.95 (0.30)	46.75 (0.29)	46.66 (0.34)	45.87 (0.33)	0.032
Sex (%)						< 0.001
Male	51.03	33.97	44.93	55.59	68.64	
Female	48.97	66.03	55.07	44.41	31.36	
Race/ethnicity (%)						< 0.001
Black	9.59	15.42	9.70	7.73	5.64	
White	72.20	66.34	72.07	74.20	76.06	
Mexican	7.18	6.99	7.23	7.28	7.21	
Other	11.04	11.25	11.00	10.80	11.09	
Education level (%)						< 0.001
Less than high school	13.09	18.44	13.95	11.42	8.75	
High school graduates	23.30	29.33	24.12	23.14	17.06	
Above high school	63.61	52.23	61.93	65.45	74.19	
Marital status (%)						< 0.001
Separated	35.23	41.90	35.69	32.91	30.57	
Married	64.77	58.10	64.31	67.09	69.43	
Family PIR (%)						< 0.001
< 1.3	18.99	27.09	19.13	16.47	13.45	
1.3–3.5	34.65	37.78	35.95	33.82	31.25	
≥ 3.5	46.37	35.13	44.92	49.71	55.31	
Uric acid (mg/dL)	5.42 (0.01)	5.35 (0.02)	5.39 (0.03)	5.46 (0.02)	5.47 (0.02)	< 0.001
eGFR (mL/(min 1.73 m^2^))	94.28 (0.29)	94.94 (0.38)	93.62 (0.37)	94.30 (0.42)	94.26 (0.41)	0.028
ALT (U/L)	25.76 (0.16)	23.57 (0.22)	25.80 (0.32)	26.92 (0.47)	26.75 (0.29)	< 0.001
Total energy intake (kcal/day)	2213.40 (8.17)	1530.28 (9.04)	2005.88 (13.09)	2388.87 (15.44)	2890.39 (18.64)	< 0.001
Caffeine (mg)	189.66 (2.68)	169.01 (3.59)	188.98 (4.04)	201.42 (4.58)	199.41 (4.54)	< 0.001
Sodium (mg)	3592.94 (15.87)	2417.50 (19.22)	3230.76 (23.73)	3883.79 (28.80)	4772.12 (33.21)	< 0.001
Hypertension (%)	35.27	37.23	37.41	35.66	31.15	< 0.001
Diabetes (%)	10.53	12.10	11.68	10.00	8.47	< 0.001
Hyperlipidemia (%)	70.39	74.05	72.19	69.25	66.32	< 0.001
CMRFs (%)	77.02	80.48	78.75	76.27	72.85	< 0.001

OBS, Oxidative Balance Score; CMRFs, cardiometabolic risk factors; PIR, poverty-to-income ratio; eGFR, estimated glomerular filtration rate; ALT, Alanine aminotransferase

**Fig. 1:**
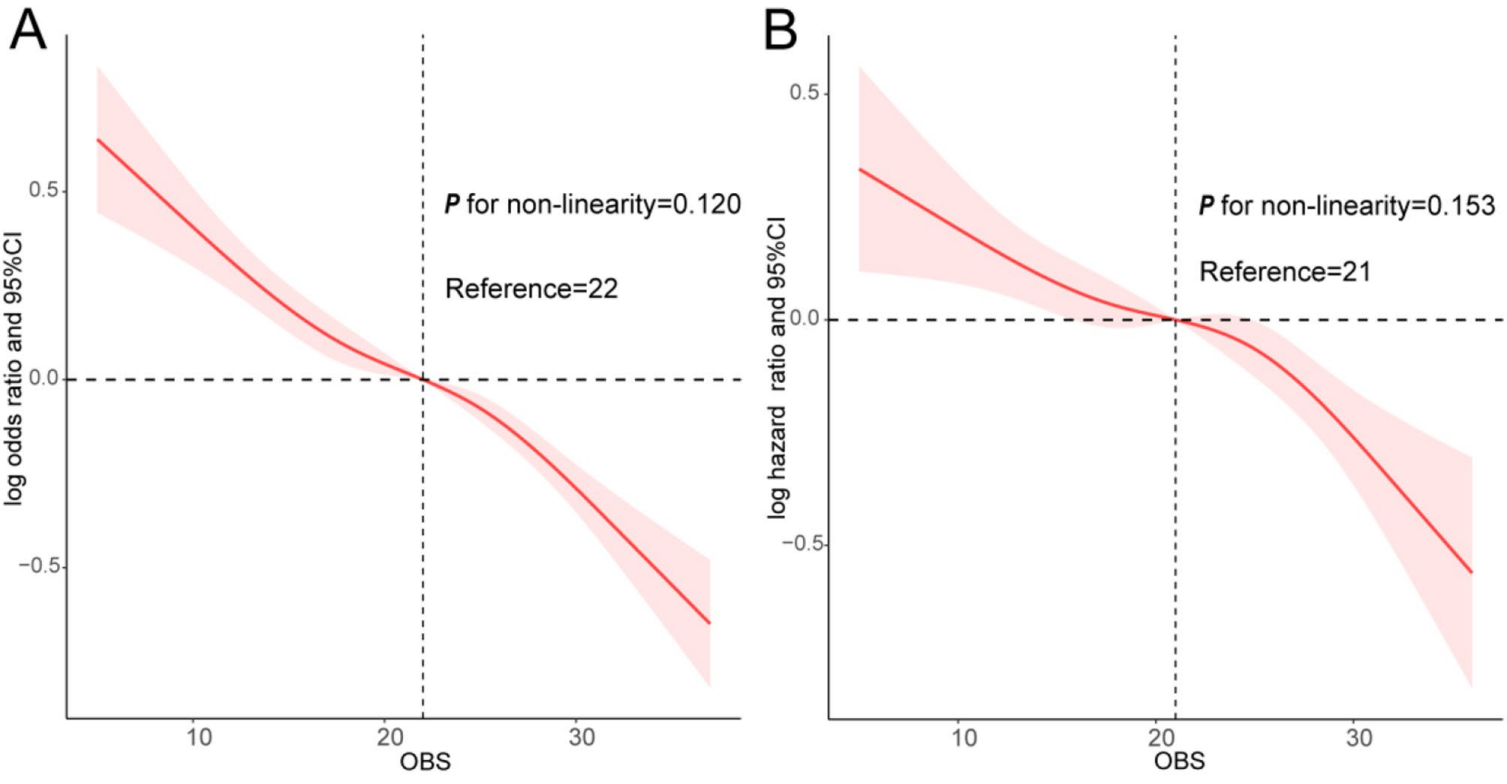
A dose-response relationship between OBS and outcomes. Associations between OBS with CMRFs (**A**) and all-cause mortality (**B**) among participants with CMRFs. OBS, Oxidative Balance Score; CMRFs, cardiometabolic risk factors

Table 2 lists the outcomes of the three multivariable logistic regression models employed to investigate the relationship between OBS, evaluated as both a continuous and quartile variable, and the risk of CMRFs. After multivariable adjustment, participants in Q2, Q3, and Q4 exhibited a risk reduction of 21%, 36%, and 46%, respectively, for CMFRs, compared to those in the lowest quartile (*P* for trend < 0.001). After adjusting for all covariates, elevated OBS was found to be significantly correlated with a decreased risk of diabetes, hypertension, and hyperlipidemia (all *P* for trend < 0.001). More specifically, there was a 33% reduction in the risk of diabetes, a 31% reduction in the risk of hypertension, and a 36% reduction in the risk of hyperlipidemia in Q4 relative to Q1. The multivariable model presented a negative association between a one-unit increment in OBS and the prevalence of CMRFs, diabetes, hypertension, and hyperlipidemia (all *P* < 0.001). Meanwhile, RCS demonstrated a significant negative linear association between OBS and CMRFs (*P* for nonlinear = 0.120, Fig. 1A). As portrayed in Table 3, after adjusting for all confounders, each 1-unit increase in dietary/lifestyle OBS was significantly and inversely linked to the risk of CMRFs, diabetes, hypertension, and hyperlipidemia (all *P* < 0.001).

**Table 2:** Associations of OBS with the risk of CMRFs

			OBS			Per one-unit increment in OBS
	Q1	Q2	Q3	Q4	*P* for trend	
Participants with any CMRFs at baseline (Participants = 29,289/ CMRFs cases = 23,190)						
Model I	Ref	0.79 (0.69, 0.91)^**†**^	0.65 (0.57, 0.74)^**‡**^	0.53 (0.47, 0.59)^**‡**^	< 0.001	0.96 (0.96, 0.97)^**‡**^
Model II	Ref	0.82 (0.71, 0.94)^*^	0.68 (0.60, 0.77)^**‡**^	0.56 (0.50, 0.63)^**‡**^	< 0.001	0.97 (0.96, 0.97)^**‡**^
Model III	Ref	0.79 (0.69, 0.92)^**†**^	0.64 (0.55, 0.73)^**‡**^	0.54 (0.47, 0.63)^**‡**^	< 0.001	0.96 (0.96, 0.97)^**‡**^
Participants with diabetes at baseline (Participants = 29,289/diabetes cases = 4233)						
Model I	Ref	0.96 (0.84, 1.09)	0.79 (0.68, 0.91)^**†**^	0.66 (0.58, 0.76)^**‡**^	< 0.001	0.98 (0.97, 0.98)^**‡**^
Model II	Ref	0.99 (0.87, 1.13)	0.82 (0.71, 0.95)^*^	0.71 (0.62, 0.82)^**‡**^	< 0.001	0.98 (0.97, 0.99)^**‡**^
Model III	Ref	0.97 (0.85, 1.10)	0.79 (0.68, 0.93)^**†**^	0.67 (0.57, 0.78)^**‡**^	< 0.001	0.97 (0.97, 0.98)^**‡**^
Participants with hypertension at baseline (Participants = 29,289/ hypertension cases = 11,736)						
Model I	Ref	0.97 (0.88, 1.07)	0.87 (0.79, 0.97)^*^	0.71 (0.63, 0.79)^**‡**^	< 0.001	0.98 (0.97, 0.98)^**‡**^
Model II	Ref	1.00 (0.91, 1.11)	0.92 (0.82, 1.02)	0.75 (0.67, 0.85)^**‡**^	< 0.001	0.98 (0.98, 0.99)^**‡**^
Model III	Ref	0.95 (0.86, 1.05)	0.84 (0.75, 0.95)^**†**^	0.69 (0.61, 0.79)^**‡**^	< 0.001	0.98 (0.97, 0.98)^**‡**^
Participants with hyperlipidemia at baseline (Participants = 29,289/hyperlipidemia cases = 20,823)						
Model I	Ref	0.83 (0.73, 0.95)^**†**^	0.70 (0.63, 0.78)^**‡**^	0.61 (0.55, 0.67)^**‡**^	< 0.001	0.97 (0.97, 0.98)^**‡**^
Model II	Ref	0.85 (0.74, 0.96)^*^	0.71 (0.64, 0.80)^**‡**^	0.63 (0.57, 0.70)^**‡**^	< 0.001	0.97 (0.97, 0.98)^**‡**^
Model III	Ref	0.84 (0.74, 0.95)^*^	0.69 (0.61, 0.79)^**‡**^	0.64 (0.56, 0.72)^**‡**^	< 0.001	0.97 (0.97, 0.98)^**‡**^

Data are expressed as the odds ratios and its 95% confidence intervals

^*^P < 0.05; ^**†**^*P* < 0.01; ^**‡**^*P* < 0.001. OBS, Oxidative Balance Score; CMRFs, cardiometabolic risk factors; ref, reference

Model I adjusted for age, sex, and race/ethnicity

Model II adjusted for model I + education level, marital status, and family poverty-to-income ratio

Model III adjusted for model II + eGFR, uric acid, ALT, total energy intake, caffeine, and sodium

**Table 3:** The association between dietary/lifestyle OBS scores and CMRFs

	Model I		Model II		Model III	
	OR (95%Cl)	*P*	OR (95%Cl)	*P*	OR (95%Cl)	*P*
Participants with any CMRFs at baseline (Participants = 29,289/ CMRFs cases = 23,190)						
Dietary OBS	0.97 (0.97, 0.98)	< 0.001	0.98 (0.97, 0.98)	< 0.001	0.97 (0.96, 0.98)	< 0.001
Lifestyle OBS	0.76 (0.74, 0.78)	< 0.001	0.76 (0.74, 0.79)	< 0.001	0.82 (0.79, 0.85)	< 0.001
Participants with diabetes at baseline (Participants = 29,289/diabetes cases = 4233)						
Dietary OBS	0.98 (0.98, 0.99)	< 0.001	0.99 (0.98, 0.99)	< 0.001	0.98 (0.97, 0.99)	< 0.001
Lifestyle OBS	0.84 (0.81, 0.87)	< 0.001	0.84 (0.82, 0.87)	< 0.001	0.85 (0.82, 0.88)	< 0.001
Participants with hypertension at baseline (Participants = 29,289/ hypertension cases = 11,736)						
Dietary OBS	0.99 (0.98, 0.99)	< 0.001	0.99 (0.99, 0.99)	0.040	0.99 (0.98, 0.99)	< 0.001
Lifestyle OBS	0.78 (0.76, 0.80)	< 0.001	0.79 (0.77, 0.81)	< 0.001	0.83 (0.80, 0.85)	< 0.001
Participants with hyperlipidemia at baseline (Participants = 29,289/hyperlipidemia cases = 20,823)						
Dietary OBS	0.98 (0.97, 0.98)	< 0.001	0.98 (0.98, 0.99)	< 0.001	0.98 (0.97, 0.99)	< 0.001
Lifestyle OBS	0.81 (0.79, 0.83)	< 0.001	0.81 (0.79, 0.83)	< 0.001	0.86 (0.84, 0.88)	< 0.001

OBS, Oxidative Balance Score; CMRFs, cardiometabolic risk factors; OR, odds ratios; 95%Cl, 95% confidence intervals

Model I adjusted for age, sex, and race/ethnicity

Model II adjusted for model I + education level, marital status, and family poverty-to-income ratio

Model III adjusted for model II + eGFR, uric acid, ALT, total energy intake, caffeine, and sodium

Noteworthily, sensitivity analysis exposed no significant change in the results, even after further adjusting for DASH and HEI-2015 scores (Supplementary Table 3). Notably, the mediation analyses unveiled significant mediating effects of GGT and WBC in the relationship between OBS and CMRFs, accounting for 11.51% and 6.90% of the total mediation proportion, respectively (*P* < 0.01, Table 4).

**Table 4:** Mediating effect by WBC and GGT on associations between CMRFs and OBS

	Direct effect	Indirect effect	Mediated proportion, %
WBC (10^9^/L)	-0.0033 (-0.0035, -0.0029)^**†**^	-0.0004 (-0.0005, -0.0003)^**†**^	11.51
GGT(IU/L)	-0.0034 (-0.0037, -0.0032)^**†**^	-0.0003 (-0.0004, -0.0001)^**†**^	6.90

^*^P < 0.05; ^**†**^*P* < 0.01; ^**‡**^*P* < 0.001

WBC, white blood cell; GGT, γ-glutamyltransferase; OBS, Oxidative Balance Score; CMRFs, cardiometabolic risk factors

The mediation analysis adjusted for age, sex, race/ethnicity, education level, marital status, family poverty-to-income ratio, eGFR, uric acid, ALT, total energy intake, caffeine, and sodium

### The association of OBS with mortality among subjects with CMRFs

Out of a total of 23,162 subjects with CMRFs in this study, 3,292 individuals succumbed to all- cause mortality, 835 individuals died due to CVD, and 789 individuals passed away as a result of cancer. RCS indicated that the correlation between OBS and all-cause mortality conformed to a dose-response pattern (*P* for nonlinear = 0.153, Fig. 1B). Table 5 presents the results of the three different Cox regression models investigating the correlation between OBS and all-cause, CVD-related, and cancer-associated deaths. In the fully adjusted model, the linear correlation between OBS and all-cause mortality continued to exhibit significance. The multivariable-adjusted hazard ratios and corresponding 95% confidence intervals for Q2, Q3, and Q4 were 0.89 (0.79, 1.00), 0.85 (0.75, 0.96), and 0.65 (0.57, 0.74) respectively, compared to Q1 (P for trend < 0.001). After full adjustment, individuals in Q4 experienced a significant decrease in the risk of CVD mortality by 40% and cancer mortality by 42% in comparison to those in Q1. Meanwhile, analyzing OBS as a continuous variable exposed that every unit increase in OBS was inversely related to all-cause, CVD-related, and cancer-related deaths (all *P* < 0.05). Table 6 illustrates similar results in the correlation analysis between dietary/lifestyle OBS and mortality. Lastly, the multivariable model suggested that each incremental unit increase in dietary/lifestyle OBS was linked to a reduced risk of all-cause, CVD-related, and cancer-related mortality (all *P* < 0.05).

**Table 5:** Association of OBS with the risk of mortality among 23,162 patients with CMRFs

			OBS			Per one-unit increment in OBS
	Q1	Q2	Q3	Q4	*P* for trend	
All-cause mortality (Number of deaths = 3292)						
Model I	Ref	0.82 (0.73, 0.91)^**‡**^	0.74 (0.65, 0.85)^**‡**^	0.54 (0.48, 0.61)^**‡**^	< 0.001	0.97 (0.96, 0.97)^**‡**^
Model II	Ref	0.89 (0.79, 0.99)^*^	0.84 (0.74, 0.96)^*^	0.63 (0.56, 0.72)^**‡**^	< 0.001	0.98 (0.97, 0.98)^**‡**^
Model III	Ref	0.89 (0.79, 0.99)^*^	0.85 (0.75, 0.96)^*^	0.65 (0.57, 0.74)^**‡**^	< 0.001	0.98 (0.97, 0.98)^**‡**^
CVD mortality (Number of deaths = 835)						
Model I	Ref	0.78 (0.63, 0.97)^*^	0.73 (0.57, 0.93)^*^	0.44 (0.33, 0.58)^**‡**^	< 0.001	0.96 (0.95, 0.97)^**‡**^
Model II	Ref	0.85 (0.69, 1.04)	0.82 (0.64, 1.05)	0.52 (0.39, 0.68)^**‡**^	< 0.001	0.97 (0.96, 0.98)^**‡**^
Model III	Ref	0.90 (0.72, 1.11)	0.91 (0.71, 1.18)	0.60 (0.44, 0.84)^**†**^	0.004	0.98 (0.97, 0.99)^*^
Cancer mortality (Number of deaths = 789)						
Model I	Ref	0.85 (0.64, 1.12)	0.77 (0.59, 1.01)	0.56 (0.42, 0.74)^**‡**^	< 0.001	0.97 (0.96, 0.98)^**‡**^
Model II	Ref	0.89 (0.68, 1.18)	0.83 (0.64, 1.09)	0.62 (0.47, 0.81)^**‡**^	< 0.001	0.97 (0.96, 0.99)^**‡**^
Model III	Ref	0.85 (0.64, 1.14)	0.79 (0.60, 1.04)	0.58 (0.42, 0.81)^**†**^	0.001	0.97 (0.95, 0.99)^**‡**^

Data are expressed as the hazard ratios and its 95% confidence intervals

^*^P < 0.05; ^**†**^*P* < 0.01; ^**‡**^*P* < 0.001. OBS, Oxidative Balance Score; CMRFs, cardiometabolic risk factors; ref, reference; CVD, cardiovascular diseases

Model I adjusted for age, sex, and race/ethnicity

Model II adjusted for model I + education level, marital status, and family poverty-to-income ratio

Model III adjusted for model II + eGFR, uric acid, ALT, total energy intake, caffeine, and sodium

**Table 6:** The association between dietary/lifestyle OBS scores with the risk of mortality among 23,162 patients with CMRFs

	Model I		Model II		Model III	
	HR (95%Cl)	*P*	HR (95%Cl)	*P*	HR (95%Cl)	*P*
All-cause mortality (Number of deaths = 3292)						
Dietary OBS	0.97 (0.96, 0.98)	< 0.001	0.98 (0.97, 0.99)	< 0.001	0.98 (0.97, 0.99)	< 0.001
Lifestyle OBS	0.89 (0.87, 0.92)	< 0.001	0.91 (0.88, 0.94)	< 0.001	0.92 (0.89, 0.95)	< 0.001
CVD mortality (Number of deaths = 835)						
Dietary OBS	0.96 (0.95, 0.98)	< 0.001	0.97 (0.96, 0.99)	< 0.001	0.98 (0.97, 0.99)	0.040
Lifestyle OBS	0.88 (0.83, 0.93)	< 0.001	0.90 (0.85, 0.96)	< 0.001	0.91 (0.85, 0.97)	0.004
Cancer mortality (Number of deaths = 789)						
Dietary OBS	0.97 (0.96, 0.99)	< 0.001	0.98 (0.96, 0.99)	0.002	0.97 (0.96, 0.99)	0.002
Lifestyle OBS	0.91 (0.85, 0.98)	0.010	0.92 (0.86, 0.99)	0.030	0.93 (0.86,0.99)	0.040

OBS, Oxidative Balance Score; CMRFs, cardiometabolic risk factors; HR, hazard ratios; 95%Cl, 95% confidence intervals; CVD, cardiovascular diseases

Model I adjusted for age, sex, and race/ethnicity

Model II adjusted for model I + education level, marital status, and family poverty-to-income ratio

Model III adjusted for model II + eGFR, uric acid, ALT, total energy intake, caffeine, and sodium

Regarding sensitivity analyses, adding DASH and HEI-2015 scores to multivariate analysis did not substantially affect the results. Nonetheless, the correlation between OBS and CVD mortality was decreased after adjusting for HEI-2015 scores (Supplementary Table 4). Besides, there was no significant change in the correlation between OBS and all-cause, CVD-related, and cancer-related deaths, even after the exclusion of individuals who died within the two-year frame or after censoring the data at a ten-year follow-up period (Supplementary Table 4).

## Discussion

This study employed cross-sectional and longitudinal designs and discovered a negative linear relationship between OBS and CMRFs in United States (US) adults, regardless of whether the former was modeled as a categorical or continuous variable. Furthermore, mediation analyses indicated that GGT and WBC have a significant mediating role in this relationship. Moreover, the cohort study showed that high OBS was linked to a decreased risk of all-causes, CVD-induced, and cancer-related deaths among individuals with CMFRs.

Previous studies have established a negative correlation between OBS and low-density lipoprotein cholesterol levels, diabetes, and new-onset hypertension [8, 13, 14]. However, the association between OBS and CMRFs risk was not analyzed, especially in large Western populations. Our study is the first to prove that increased OBS was related to a decreased risk of CMFRs in US adults. Multiple findings have explored the effect of dietary antioxidants on these three CMRFs by other evaluation methods, determining that individuals with elevated levels of dietary antioxidant capacity exhibit a reduced likelihood of developing hypertension, diabetes, and dyslipidemia in comparison to those with decreased levels of dietary antioxidant capacity [25–28]. Moreover, a small sample study based on the Chinese elderly population has demonstrated a negative relationship between lifestyle OBS and CMFRs [11]. Our study revealed an inverse relationship between dietary OBS and lifestyle OBS and the prevalence of CMRFs. Hence, we postulate that OBS can serve as a valuable tool to guide the general population toward adopting healthy diets and positive lifestyle patterns to reduce the occurrence of CMRFs.

Previous research has demonstrated a correlation between lower OBS and unfavorable prognostic outcomes. A study conducted among Spanish university students suggested that a higher OBS may decrease the risk of all-cause mortality, including deaths related to CVD and cancer [16]. Similarly, a study implemented in the stroke belt region of the United States found an inverse relationship between high OBS and all-cause mortality in individuals aged 45 and older [17]. As is well documented, the prognosis of CMRFs patients is poor. Therefore, early intervention is crucial in CMRFs patients. In this study, high OBS was found to be related to a decreased risk of all-cause, CVD-related, and cancer-related mortality among individuals with CMRFs. Wang et al. demonstrated that dietary OBS was negatively linked to deaths from all causes and CVD [29]. We demonstrated that individuals with CMRFs who are exposed to higher levels of dietary or lifestyle OBS may potentially exhibit a reduced risk of mortality. In summary, the current research findings have significant implications for public health, particularly in developing strategies to reduce the risk of CMRFs and improve the prognosis of CMRFs patients.

Consistent with the observations of prior studies [13, 19, 30], the dietary prooxidants (iron and total fat) exhibited an upward trend with a rise in OBS, which contradicted the OBS assignment scheme. This discrepancy might be ascribed to higher total energy intake. While a single antioxidant or prooxidant may be involved in the etiology and development of CMRFs, it is crucial to consider their antagonistic or synergistic effects. Therefore, OBS was used as a comprehensive assessment tool to evaluate oxidative balance in individuals and explore its impact on CMRFs. We observed a dose-response relationship between OBS and the risk and prognosis of CMRFs. Consistent with our results, a cohort study reported a significant inverse dose-response correlation between elevated OBS and the occurrence of new-onset hypertension [14]. Similarly, another study reported a progressive decline in the risk of all-cause mortality as the intake of dietary OBS increased [29]. In summary, our findings provide compelling evidence to endorse a public health recommendation that dietary and lifestyle antioxidants can effectively mitigate the risk of CMRFs and enhance the prognosis of patients.

The exact mechanism by which OBS affects CMRFs remains elusive, and oxidative stress may play an important role. Oxidative stress refers to the disruption of the equilibrium between intracellular antioxidants and prooxidants, frequently accompanied by the accumulation of reactive oxygen species (ROS) [31]. Firstly, ROS drives insulin resistance and lipid peroxidation through multiple mechanisms, thereby exerting adverse effects on blood pressure, glucose, and lipid metabolism and increasing the risk of CVD [31–35]. Secondly, ROS contributes to the development of hypertension by inducing endothelial damage, vascular dysfunction and remodeling, and sympathetic nervous system excitation [36]. Thirdly, the occurrence of oxidative stress triggers a reduction in nitric oxide levels, leading to insulin resistance and endothelial dysfunction, thereby increasing the risk of diabetes and hypertension [37, 38]. In conjunction with oxidative stress, activation of the inflammatory response also plays a major role in the pathophysiology of CMRFs. Inflammatory mediators stimulate immune cell activation within target organs, thereby exacerbating vascular dysfunction, remodeling, and fibrosis, collectively leading to elevated blood pressure [39]. Chronic low-grade inflammation is causally correlated with insulin resistance, as well as the activation and infiltration of immune cells, thereby promoting the occurrence and development of diabetes [40, 41]. CMFRs in turn increase ROS production and trigger inflammatory responses [42, 43]. Thus, the interaction between oxidative stress, inflammatory response, and CMRFs is multidirectional and complex. Previous findings have established that OBS functions as a biomarker for oxidative stress and inflammation. These researches have yielded evidence indicating a negative correlation between elevated OBS levels and decreased levels of inflammatory markers such as C-reactive protein (CRP) and WBC [8], as well as oxidative stress biomarkers, specifically serum GGT [9]. Overall, it is reasonable to speculate that inflammatory response and oxidative stress may act as potential mediators between OBS and CMRFs. Our research confirms the above viewpoint and reveals that the relationship between OBS and CMRFs is partially mediated by GGT and WBC.

Despite the support from a substantial sample size and a long-term NHANES follow-up, certain limitations in our research should be acknowledged. To begin, the cross-sectional nature of our study inherently limited our ability to establish causal relationships between OBS and CMFRs. Secondly, self-reported questionnaires may lead to biases in assessing dietary OBS. Thirdly, our analysis only considered baseline OBS, without considering potential changes in diet and lifestyle during follow-up. Regrettably, the limitations of NHANES precluded dynamic adjustments in OBS. Fourth, there exist other inflammatory indicators, such as CRP, serum amyloid A, interleukin, and cytokines, as well as oxidative stress indicators like oxidized low-density lipoprotein, glutathione, and malondialdehyde [44, 45]. The constraints of the NHANES database hinder our ability to delve deeper into the potential mediating function of these biomarkers in the relationship between OBS and CMRFs. Finally, despite conducting a multivariate analysis, the possibility of selection bias influencing our findings cannot be excluded.

## Conclusion

This cross-sectional and cohort study suggests that an increase in OBS among US adults may reduce the risk of CMRFs and improve the prognosis of CMRFs patients. In addition, our study reveals that the GGT and WBC may play a potential mediating role in the association between OBS and CMRFs.

### Electronic supplementary material

Below is the link to the electronic supplementary material.

Supplementary Material 1

Supplementary Material 2

Supplementary Material 3

## Data Availability

All data are publicly available and can be accessed at the NHANES (https://www.cdc.gov/nchs/nhanes/index.htm).
