# Supplementary material for: The cross-sectional correlation between the oxidative balance score and cardiometabolic risk factors and its potential correlation with longitudinal mortality in patients with cardiometabolic risk factors
Source: BMC Public Health. 2024 May 30;24:1452. doi: 10.1186/s12889-024-18967-z (PMC11140939; doi:10.1186/s12889-024-18967-z)
Supplement: Supplementary file 1 — Supplementary Material 1 [file 12889_2024_18967_MOESM1_ESM.docx]

Supplementary Table 1 The assessment of OBS.

| OBS components | Property | Assignment Scheme |
| --- | --- | --- |
| Dietary OBS components |  |  |
| Dietary fiber (g/d) | A | 0 = Tertile 1 (low), 1 = Tertile 2 (intermediate), 2 = Tertile 3 (high) |
| Carotene (RE/d) | A | 0 = Tertile 1 (low), 1 = Tertile 2 (intermediate), 2 = Tertile 3 (high) |
| Riboflavin (mg/d) | A | 0 = Tertile 1 (low), 1 = Tertile 2 (intermediate), 2 = Tertile 3 (high) |
| Niacin (mg/d) | A | 0 = Tertile 1 (low), 1 = Tertile 2 (intermediate), 2 = Tertile 3 (high) |
| Vitamin B6 (mg/d) | A | 0 = Tertile 1 (low), 1 = Tertile 2 (intermediate), 2 = Tertile 3 (high) |
| Total folate (mcg/d) | A | 0 = Tertile 1 (low), 1 = Tertile 2 (intermediate), 2 = Tertile 3 (high) |
| Vitamin B12 (mcg/d) | A | 0 = Tertile 1 (low), 1 = Tertile 2 (intermediate), 2 = Tertile 3 (high) |
| Vitamin C (mg/d) | A | 0 = Tertile 1 (low), 1 = Tertile 2 (intermediate), 2 = Tertile 3 (high) |
| Vitamin E (mg/d) | A | 0 = Tertile 1 (low), 1 = Tertile 2 (intermediate), 2 = Tertile 3 (high) |
| Calcium (mg/d) | A | 0 = Tertile 1 (low), 1 = Tertile 2 (intermediate), 2 = Tertile 3 (high) |
| Magnesium (mg/d) | A | 0 = Tertile 1 (low), 1 = Tertile 2 (intermediate), 2 = Tertile 3 (high) |
| Zinc (mg/d) | A | 0 = Tertile 1 (low), 1 = Tertile 2 (intermediate), 2 = Tertile 3 (high) |
| Copper (mg/d) | A | 0 = Tertile 1 (low), 1 = Tertile 2 (intermediate), 2 = Tertile 3 (high) |
| Selenium (mcg/d) | A | 0 = Tertile 1 (low), 1 = Tertile 2 (intermediate), 2 = Tertile 3 (high) |
| Total fat (g/d) | P | 0 = Tertile 3 (high), 1 = Tertile 2 (intermediate), 2 = Tertile 1 (low) |
| Iron (mg/d) | P | 0 = Tertile 3 (high), 1 = Tertile 2 (intermediate), 2 = Tertile 1 (low) |
| Lifestyle OBS components |  |  |
| Physical activity (METs-h/week) | A | 0=low (<7.5 METs-h/wk), 1=moderate (7.5-30 METs-h/wk), 2=high  (>30 METs-h/wk) |
| Alcohol (g/d) | P | 0 = heavy drinker (≥30 g/day for males, ≥20 g/day for females), 1= mild-to-moderate drinker (<30 g/day for males, <20 g/day for females), 2 = non-drinker |
| Body mass index (kg/m^2^ ) | P | 0=obesity (≥30 kg/m^2^), 1= overweight (25-29.9 kg/m^2^), 2=normal (<25 kg/m^2^) |
| Cotinine (ng/mL) | P | 0 = Tertile 3 (high), 1 = Tertile 2 (intermediate), 2 = Tertile 1 (low) |

OBS, Oxidative Balance Score; P, pro-oxidant; A, antioxidant.

Supplementary Table 2 Individual components of the score by oxidative balance score quartiles .

| Characteristics | Total | Q1 | Q2 | Q3 | Q4 | *P* |
| --- | --- | --- | --- | --- | --- | --- |
|  | N=29289 | N=8298 | N=7350 | N=6574 | N=7067 |  |
| Dietary fiber (g/d) | 17.26 (0.12) | 9.46 (0.07) | 14.31 (0.09) | 18.58 (0.12) | 26.10 (0.18) | <0.001 |
| Carotene (RE/d) | 242.56 (5.06) | 117.71 (3.85) | 192.50 (5.86) | 267.89 (12.20) | 383.28 (11.23) | <0.001 |
| Riboflavin (mg/d) | 2.23 (0.01) | 1.32 (0.01) | 1.93 (0.01) | 2.41 (0.02) | 3.21 (0.03) | <0.001 |
| Niacin (mg/d) | 25.93 (0.12) | 15.65 (0.11) | 22.67 (0.19) | 28.25 (0.21) | 36.51 (0.26) | <0.001 |
| Vitamin B6 (mg/d) | 2.13 (0.01) | 1.15 (0.01) | 1.79 (0.02) | 2.31 (0.02) | 3.19 (0.03) | <0.001 |
| Total folate (mcg/d) | 416.75 (2.58) | 232.66 (1.63) | 344.15 (2.34) | 447.12 (3.45) | 629.15 (4.53) | <0.001 |
| Vitamin B12 (mcg/d) | 5.38 (0.06) | 2.73 (0.03) | 4.41 (0.05) | 5.88 (0.10) | 8.29 (0.16) | <0.001 |
| Vitamin C (mg/d) | 88.06 (1.03) | 46.65 (0.88) | 70.78 (1.16) | 93.42 (1.34) | 137.98 (1.89) | <0.001 |
| Vitamin E (mg/d) | 8.41 (0.07) | 4.53 (0.04) | 6.85 (0.06) | 8.96 (0.08) | 12.99 (0.13) | <0.001 |
| Calcium (mg/d) | 960.30 (5.49) | 556.29 (4.20) | 812.74 (4.86) | 1036.87 (8.19) | 1407.03 (9.82) | <0.001 |
| Magnesium (mg/d) | 307.17 (1.71) | 177.95 (1.06) | 260.80 (0.92) | 332.70 (1.46) | 448.41 (2.27) | <0.001 |
| Zinc (mg/d) | 12.05 (0.07) | 7.01 (0.07) | 10.24 (0.08) | 13.22 (0.14) | 17.42 (0.13) | <0.001 |
| Copper (mg/d) | 1.34 (0.01) | 0.78 (0.01) | 1.13 (0.01) | 1.44 (0.01) | 1.97 (0.02) | <0.001 |
| Selenium (mcg/d) | 114.75 (0.51) | 72.86 (0.46) | 101.85 (0.69) | 124.05 (0.78) | 157.67 (1.11) | <0.001 |
| Total fat (g/d) | 82.96 (0.38) | 56.38 (0.39) | 75.43 (0.54) | 89.98 (0.68) | 108.62 (0.88) | <0.001 |
| Iron (mg/d) | 15.63 (0.08) | 9.26 (0.06) | 13.18 (0.08) | 16.76 (0.12) | 22.84 (0.16) | <0.001 |
| Physical activity (METs-h/week) | 53.64 (1.05) | 45.05 (1.52) | 48.97 (1.41) | 54.02 (1.59) | 65.61 (1.64) | <0.001 |
| Alcohol (g/d) | 10.24 (0.26) | 8.29 (0.37) | 10.58 (0.41) | 12.37 (0.52) | 9.89 (0.53) | <0.001 |
| Body mass index (kg/m^2^) | 28.45 (0.08) | 29.42 (0.11) | 28.68 (0.12) | 28.46 (0.12) | 27.33 (0.11) | <0.001 |
| Cotinine (ng/mL) | 55.60 (1.59) | 83.88 (2.56) | 58.19 (2.51) | 48.12 (2.22) | 33.25 (1.84) | <0.001 |

Supplementary Table 3 Associations of OBS with the risk of CMRFs (Participants=29289/ CMRFs cases=23190).

|  | OBS | | | | | Per one-unit increment in OBS |
| --- | --- | --- | --- | --- | --- | --- |
|  | Q1 | Q2 | Q3 | Q4 | P for trend |  |
| Participants with any CMRFs at baseline (Participants=29289/ CMRFs cases=23190) | | | | | | |
| Model III+DASH | Ref | 0.81 (0.70, 0.94)^*^ | 0.66 (0.57, 0.77)**^‡^** | 0.59 (0.50, 0.69)**^‡^** | <0.001 | 0.97 (0.96, 0.97)**^‡^** |
| Model III+HEI-2015 | Ref | 0.85 (0.73, 0.98)^*^ | 0.71 (0.61, 0.82)**^‡^** | 0.65 (0.55, 0.77)**^‡^** | <0.001 | 0.97 (0.96, 0.98)**^‡^** |
| Participants with diabetes at baseline (Participants=29289/diabetes cases=4233) | | | | | | |
| Model III+DASH | Ref | 0.98 (0.86, 1.12) | 0.82 (0.70, 0.96)^*^ | 0.71 (0.59, 0.84)**^‡^** | <0.001 | 0.98 (0.97, 0.99)**^‡^** |
| Model III+HEI-2015 | Ref | 0.98 (0.86, 1.12) | 0.81 (0.70, 0.95)^*^ | 0.70 (0.58, 0.83)**^‡^** | <0.001 | 0.98 (0.97, 0.99)**^‡^** |
| Participants with hypertension at baseline (Participants=29289/ hypertension cases=11736) | | | | | | |
| Model III+DASH | Ref | 0.96 (0.87, 1.06) | 0.86 (0.76, 0.97)^*^ | 0.71 (0.63, 0.81)**^‡^** | <0.001 | 0.98 (0.97, 0.98)**^‡^** |
| Model III+HEI-2015 | Ref | 0.97 (0.87,1.07) | 0.87 (0.77, 0.99)^*^ | 0.73 (0.64, 0.84)**^‡^** | <0.001 | 0.98 (0.97, 0.99)**^‡^** |
| Participants with hyperlipidemia at baseline (Participants=29289/hyperlipidemia cases=20823) | | | | | | |
| Model III+DASH | Ref | 0.85 (0.75, 0.97)^*^ | 0.72 (0.63, 0.82)**^‡^** | 0.68 (0.58, 0.78)**^‡^** | <0.001 | 0.97 (0.97, 0.98)**^‡^** |
| Model III+HEI-2015 | Ref | 0.89 (0.78, 1.01) | 0.76 (0.67, 0.88)**^‡^** | 0.75 (0.65, 0.87)**^‡^** | <0.001 | 0.98 (0.97, 0.99)**^‡^** |

^*^ *P*<0.05; **^†^** *P*<0.01; **^‡^** *P*<0.001.

Data are expressed as the odds ratios and its 95% confidence intervals.

OBS, Oxidative Balance Score; CMRFs, cardiometabolic risk factors; ref, reference; DASH, Dietary Approaches to Stop Hypertension; HEI, Healthy Eating Index.

Model III adjusted for age, sex, race/ethnicity, education level, marital status, family poverty-to-income ratio, eGFR, uric acid, ALT, total energy intake, caffeine, and sodium.

Supplementary Table 4 Association of OBS with the risk of mortality among 23162 patients with CMRFs.

|  | OBS | | | | | Per one-unit increment in OBS |
| --- | --- | --- | --- | --- | --- | --- |
|  | Q1 | Q2 | Q3 | Q4 | P for trend |  |
| All-cause mortality (Number of deaths=3292) | | | | | | |
| Model III+DASH | Ref | 0.89 (0.79, 1.00) | 0.85 (0.74, 0.98)^*^ | 0.65 (0.56, 0.76)**^‡^** | <0.001 | 0.98 (0.97, 0.99)**^‡^** |
| Model III+HEI-2015 | Ref | 0.93 (0.83, 1.05) | 0.93 (0.81, 1.06) | 0.74 (0.62, 0.87)**^‡^** | <0.001 | 0.98 (0.98, 0.99)**^‡^** |
| Model III with restrictions | | | | | | |
| Excluding events within first 2 years | Ref | 0.89 (0.79, 1.01) | 0.88 (0.77, 1.00) | 0.66 (0.58, 0.75)**^‡^** | <0.001 | 0.98 (0.97, 0.99)**^‡^** |
| Censoring at 10 years follow-up | Ref | 0.93 (0.81, 1.07) | 0.79 (0.67, 0.93)^*^ | 0.65 (0.54, 0.78)**^‡^** | <0.001 | 0.97 (0.97, 0.98)**^‡^** |
| CVD mortality (Number of deaths=835) | | | | | | |
| Model III+DASH | Ref | 0.90 (0.72, 1.13) | 0.92 (0.69, 1.23) | 0.61 (0.43, 0.86)**^†^** | <0.001 | 0.98 (0.96, 0.99)^*^ |
| Model III+HEI-2015 | Ref | 0.94 (0.75, 1.18) | 1.00 (0.76, 1.33) | 0.69 (0.49, 0.99)^*^ | <0.001 | 0.99 (0.97, 1.00) |
| Model III with restrictions | | | | | | |
| Excluding events within first 2 years | Ref | 0.89 (0.72, 1.10) | 0.97 (0.74, 1.26) | 0.53 (0.39, 0.71)**^‡^** | <0.001 | 0.98 (0.97, 0.99)**^†^** |
| Censoring at 10 years follow-up | Ref | 0.93 (0.68, 1.28) | 0.98 (0.71, 1.34) | 0.61 (0.38, 0.98)^*^ | <0.001 | 0.98 (0.96, 0.99)^*^ |
| Cancer mortality (Number of deaths=789) | | | | | | |
| Model III+DASH | Ref | 0.86 (0.64, 1.16) | 0.81 (0.61, 1.07) | 0.60 (0.42, 0.87)^*^ | <0.001 | 0.97 (0.95, 0.99)**^†^** |
| Model III+HEI-2015 | Ref | 0.89 (0.66, 1.19) | 0.85 (0.63, 1.15) | 0.66 (0.45, 0.95)^*^ | <0.001 | 0.98 (0.96, 0.99)^*^ |
| Model III with restrictions | | | | | | |
| Excluding events within first 2 years | Ref | 0.88 (0.65, 1.20) | 0.85 (0.64, 1.14) | 0.62 (0.44, 0.89)^*^ | 0.012 | 0.98 (0.96, 0.99)^*^ |
| Censoring at 10 years follow-up | Ref | 0.85 (0.61, 1.20) | 0.75 (0.53, 1.05) | 0.64 (0.45, 0.91)^*^ | <0.001 | 0.97 (0.95, 0.99)**^†^** |

^*^ *P*<0.05; **^†^** *P*<0.01; **^‡^** *P*<0.001.

Data are expressed as the hazard ratios and its 95% confidence intervals.

OBS, Oxidative Balance Score; CMRFs, cardiometabolic risk factors; DASH, Dietary Approaches to Stop Hypertension; HEI, Healthy Eating Index; ref, reference; CVD, cardiovascular diseases. Model III adjusted for age, sex, race/ethnicity, education level, marital status, family poverty-to-income ratio, eGFR, uric acid, ALT, total energy intake, caffeine, and sodium.
