# Supplementary material for: The cross-sectional correlation between the oxidative balance score and cardiometabolic risk factors and its potential correlation with longitudinal mortality in patients with cardiometabolic risk factors
Source: BMC Public Health. 2024 May 30;24:1452. doi: 10.1186/s12889-024-18967-z (PMC11140939; doi:10.1186/s12889-024-18967-z)
Supplement: Supplementary file 2 — Supplementary Material 2 [file 12889_2024_18967_MOESM2_ESM.docx]

**The definition of diabetes, hypertension, and hyperlipidemia.**

Diabetes was determined by self-reported diagnosis, using diabetes medications, hemoglobin A1c ≥6.5%, or fasting plasma glucose ≥126 mg/dL. Hypertension was considered by self-reported diagnosis, using antihypertension medications, or systolic/diastolic blood pressure ≥140/90 mm Hg. Hyperlipidemia was diagnosed by self-reported diagnosis, triglyceride ≥150 mg/dL, total cholesterol ≥200 mg/dL, high-density lipoprotein cholesterol <40 mg/dL for males or <50 for females, or low-density lipoprotein cholesterol ≥130 mg/dL.

**OBS assessment**

The dietary intake of each participant was determined by two 24-hour dietary recall surveys whenever data for two days were available. In cases where only one day of dietary recall data was available, data from the first day was used for calculation purposes. The participants in this study were allocated points ranging from 0 to 2 for dietary antioxidants based on the distribution of tertiles within the study population. Tertile 1 to Tertile 3 were assigned ascending point values. Conversely, a reversed scoring system was utilized for dietary pro-oxidants. Participants in Tertile 1 received the highest score of 2 points, whereas those in Tertile 3 obtained the lowest score of 0 points. Lifestyle component scores were computed using the following methodology: cotinine levels were employed to estimate smoking status and allocate scores, ranging from 0 to 2, to Tertile 3 through Tertile 1, respectively. Body Mass Index (BMI) was stratified into three categories: 2 points (<25 kg/m^2^), 1 point (25-29.9 kg/m^2^), or 0 points (≥30 kg/m^2^). Non-drinkers participants were allocated 2 points, whereas individuals who consumed alcohol within the range of 20 g/day for females and 30 g/day for males were awarded 1 point. Conversely, those who exceeded the aforementioned limits, that is, consuming over 20 g/day for females and over 30 g/day for males, were assigned 0 points [13]. Physical activity was evaluated as weekly metabolic equivalents (METs) and awarded a score of 0 (<7.5 METs-h/wk), 1 (7.5-30 METs-h/wk), and 2 (>30 METs-h/wk) [13].

**The calculation for eGFR**

The calculation for eGFR used as the formula: 141×min (SCr/κ, 1)α×max (SCr/κ, 1)−1.209×0.993Age×1.018 if female×1.159 if black, where SCr represents serum creatinine, κ is 0.7 for females and 0.9 for males, α is −0.329 for females and −0.411 for males, min denotes the lesser value between SCr/κ or 1, and max signifies the greater value between SCr/κ or 1.
