# Supplementary material for: The cross-sectional correlation between the oxidative balance score and cardiometabolic risk factors and its potential correlation with longitudinal mortality in patients with cardiometabolic risk factors
Source: BMC Public Health. 2024 May 30;24:1452. doi: 10.1186/s12889-024-18967-z (PMC11140939; doi:10.1186/s12889-024-18967-z)
Supplement: Supplementary file 3 — Supplementary Material 3 [file 12889_2024_18967_MOESM3_ESM.docx]

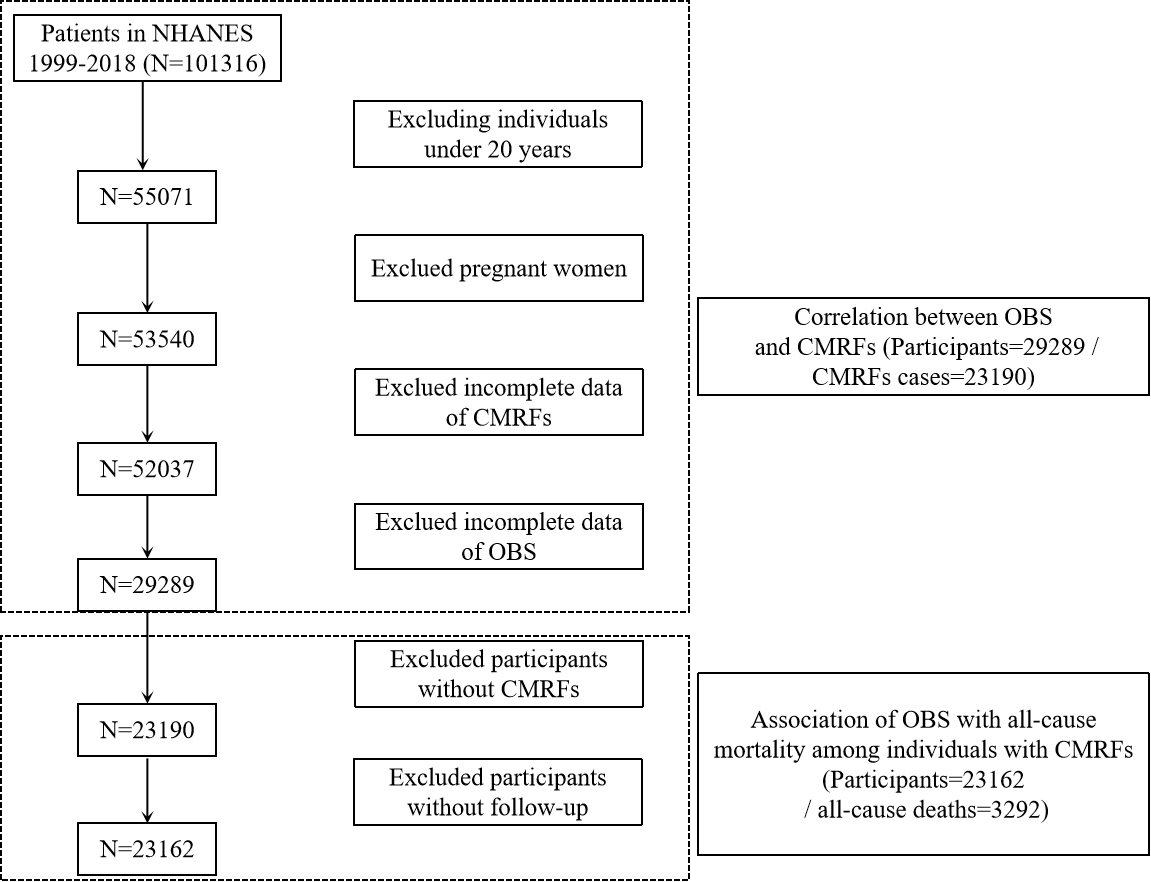


Supplementary Figure 1 Flowchart of the sample selection from NHANES 1999–2018. NHANES, National Health and Nutrition Examination Survey; Cardiometabolic risk factors, CMRFs; Oxidative balance score, OBS.
